# Gene Silencing of *Olfactory Receptor Coreceptor* by Systemic RNA Interference in *Callosobruchus maculatus*

**DOI:** 10.1007/s10886-025-01553-x

**Published:** 2025-01-24

**Authors:** Kenji Shimomura, Keito Sakita, Takehito Terajima, Motohiro Tomizawa

**Affiliations:** Department of Chemistry for Life Sciences and Agriculture, Faculty of Life Sciences, Tokyo University of Agriculture, Tokyo, Japan

**Keywords:** Seed beetle, *Callosobruchus maculatus*, RNA interference, Olfactory receptor-coreceptor

## Abstract

Seed beetles are pernicious pests of leguminous seeds and are distributed globally. They cause great economic losses, particularly in developing countries. Of this genus, the cowpea weevil (*Callosobruchus maculatus*) is the most destructive and common species of this beetle. However, there are no effective and sustainable control strategies available for this species. Nevertheless, sustainable pest management strategies using sex pheromone compounds have been proposed, as *C. maculatus* uses species-specific sex pheromone signals for pre-mating isolation. Therefore, this study aimed to investigate the attractive capacity of male *C. maculatus* after RNA interference (RNAi)-based gene knockdown of olfactory receptor coreceptor (*Cmac\Orco*). The RNAi effect showed more than 90% reduction in transcripts, and a behavioral bioassay using a Y-tube olfactometer indicated that knocking down males impaired sexual attraction toward females, which would be a useful tool for further screening of target molecules for odorant chemical communication.

## Introduction

The detection of odorant signals provides animals and insects with crucial information relating to the environment, and detection of mates, hosts, food resources, predators, and pathogens (Andersson et al. 2015). In insects, species-specific sex pheromone signals are crucial for pre-mating isolation and speciation, any minor variations in sex pheromones can alter their effectiveness (Symonds and Elgar 2008). However, it is unclear as to how these altered sex pheromones are emitted and received; and how these signals concertedly evolve, as signal production and reception is rarely linked (Niehuis et al. 2013).

In the antennae of insects, odorant detection occurs through the dendritic membrane of olfactory sensory neurons (OSNs), where a receptor assembles a heteromeric tetramer formed by the olfactory receptor (OR) and olfactory receptor coreceptor (Orco); the amino acid sequences of Orcos are highly conserved in insects. Therefore, Orco mutants and/or the knock down of *Orco* in insects’ result in failure to detect odorants (Stengl and Funk 2013).

Coleoptera is the largest and most diverse insect order in the animal kingdom to date. *Callosobruchus* seed beetles (Coleoptera: Chysomelidae: Bruchinae) are pernicious pests of stored grain pulses that are distributed globally, with high prevalence in tropical and subtropical areas. Among these beetles, the cowpea weevil, *Callosobruchus maculatus*, is the most prominent pest species. The females lay their eggs on the surface of a seed, these eggs then hatch as first-instar larva which enter the seed and continuously feed on the embryo and endosperm. These larvae then continue their life cycle within the seed by transforming into the pupa stage and then emerging from the seed as an adult. The loss of storage pulses by *C. maculatus* is estimated to be 10–20%, the effective and sustainable pest control method alternative to traditional management has been desired (Kalpna et al. 2022).

Sex attractant pheromones, (*Z*)-3-methyl-2-heptenoic acid, (*Z*)-3-methyl-3-heptenoic acid, (*E*)-3-methyl-2-heptenoic acid, (*E*)-3-methyl-3-heptenoic acid, and 3-methyleneheptanoic acid, and contact sex pheromones, 2,6-dimethyloctanedioic acid and azelaic acid (nonanedioic acid) with a neutral mixture of C_27_–C_35_ straight-chain and methyl-branched saturated hydrocarbon compounds were previously identified from *C. maculatus* for the potential of the semiochemical-based pest control (Phillips et al. 1996; Nojima et al. 2007). Moreover, antennal transcriptome analysis was conducted on the beetle to identify chemosensory-related genes (Tanaka et al. 2022), but specific ligands remain to be identified for each gene.

RNA interference (RNAi) is a post-transcriptional mRNA-silencing mechanism in which double-stranded RNA (dsRNA) directs the cleavage of complementary mRNA. Moreover, RNAi is an endogenous cellular process that is widely conserved among eukaryotic organisms. In insects, RNAi is a powerful reverse genetics tool for uncovering gene functions as well as delivery methods, such as microinjection, feeding, and nanoparticle spray-induction. In Coleoptera, RNAi has been applied for dsRNA-based gene silencing, as RNAi knockdown is highly efficient and systemic (Zhu and Palli 2020). For alternative pest control strategy based on olfactory-system disruption, RNAi-based knockdown of *Orco* gene has been performed (e.g. *Rhynchophorus ferrugineus*: Soffan et al. 2016).

In this study, we aimed to successfully knockdown *Cmac\Orco* using RNAi to clarify the malfunctioning effect of *Cmac\Orco* on sexual attraction. The results would contribute to understanding the pheromonal communication system and subsequent development of semiochemical-based management to the beetle.

## Methods and Materials

*Insects.* Laboratory colonies of *C. maculatus* were used in this study. Beetles were reared on *Vigna angularis* in a plastic container which was maintained at 28 °C in a dark incubator.

*Cloning of the C. maculatus Orco gene.* For In-Fusion cloning of *Cmac\Orco* gene, 200 antennae were collected from adult beetles and homogenized using a Biomasher II (Nippi Inc., Tokyo, Japan). Total RNA was extracted using a ReliaPrep RNA Cell Miniprep System (Promega Corporation, Madison, Wl, USA) and first strand complementary DNA (cDNA) was synthesized with ReverTra Ace -α- (Toyobo Co., Ltd., Osaka, Japan) according to the manufacturer’s methods. Full length *Cmac\Orco* gene was amplified by PCR with a KOD Plus Neo (Toyobo) using specific primers based on the sequence (Tanaka et al. 2022): forward 5’-TCGGTACCCGGGGATCATGATGAAATTCAAGGTAGCAGG-3’, reverse 5’-TCGACTCTAGAGGATCCTATTTGAGTTGAACCAGCACC-3’. The PCR was performed on a T100 thermal cycler (Bio-Rad, Hercules, CA, USA) under the following conditions: 94 °C for 2 min, followed by 35 cycles of 98 °C for 10 s, 58 °C for 30 s, and 68 °C for 1.5 min. After agarose-gel electrophoresis and purification using NucleoSpin Gel and PCR Clean-up (Takara Bio Inc., Shiga, Japan), the PCR product was assembled with a linearized pUC19 plasmid vector using the In-Fusion HD cloning kit (Takara Bio). The assembled vector was then transformed into ECOS Competent *Escherichia coli* DH5α cell (Nippon Genetics, Tokyo, Japan). Plasmid DNA was purified using the FastGene Plasmid Mini kit (Nippon Genetics) and sequenced (Fasmac Co., Ltd., Kanagawa, Japan). The sequence data has been deposited in GenBank (PQ660494).

*RNAi-mediated Cmac\Orco knock down using the microinjection method.* The partial *Cmac\Orco* gene containing a T7 promoter sequence at the 5’ end was amplified with primers: forward 5’-TAATACGACTCACTATAGGGTTGCTTGTGAACAGTTGCAGCA-3’, reverse 5’-TAATACGACTCACTATAGGGACCAATGGCAACTGTATGCTGC-3’, using KOD Plus Neo under the following conditions: 94 °C for 2 min, followed by 40 cycles at 98 °C for 10 s, 58 °C for 30 s, and 68 °C for 1 min. The amplified template was used to synthesize dsRNA using the RiboMAX Large Scale RNA Production System T7 (Promega). For the control, we prepared the following three lines: non-injection, H_2_O injection, and *dsEGFP* injection. *EGFP* dsRNA was synthesized from the pEGFP-C1 plasmid (Takara Bio USA, Inc., Mountain View, CA, USA). RNase free water was used for the dilution of the dsRNA solution and adjustment of the concentration to 2 µg/µL.

The beetles were carefully extracted from the seeds at the pupal stage, the collected pupae were set on the stage of a microscope and dsRNA was microinjected into the dorsal abdominal part of the pupa using a micromanipulator (Narishige, Tokyo, Japan). Afterwards, the pupae were individually inserted into a 24-well cell culture plastic plate and placed at incubator for 7–10 d without seeds. Newly emerged males were collected and placed separately in a glass tube (12 mm diameter, 30 mm length), both ends of the glass tube were covered by cotton balls and incubated further for an olfactometer assay with the same condition above.

*Olfactometer assay.* A Y-tube olfactometer was used, as previously reported (Shimomura et al. 2008). Briefly, 15 virgin females of *C. maculatus* were placed in a small metal mesh basket (10 mm diameter, 30 mm length) which was inserted into one side arm; on the other side arm an empty basket was inserted. Clean air was passed through the activated charcoal from the side arms into the main tube at a rate of 1 L/min. After the cotton balls were removed, the glass tube containing the male was placed into the end of the main tube. The assay was performed for 2 min or until the male contacted either basket, and the amount of the time elapsed until each male made contact with the female basket was measured. Sixty male beetles were used for each treatment group. Once the assay was completed, the antennae were removed immediately from the males and preserved at -80 °C until total RNA extraction.

*Quantitative reverse transcription PCR (qRT-PCR) analysis.* The 60 male antennae were divided to 3 groups (20 male antennae each group). Total RNA was extracted from the antennae, and cDNA synthesis and qRT-PCR were performed as previously reported (Tanaka et al. 2022).

**Fig. 1:**
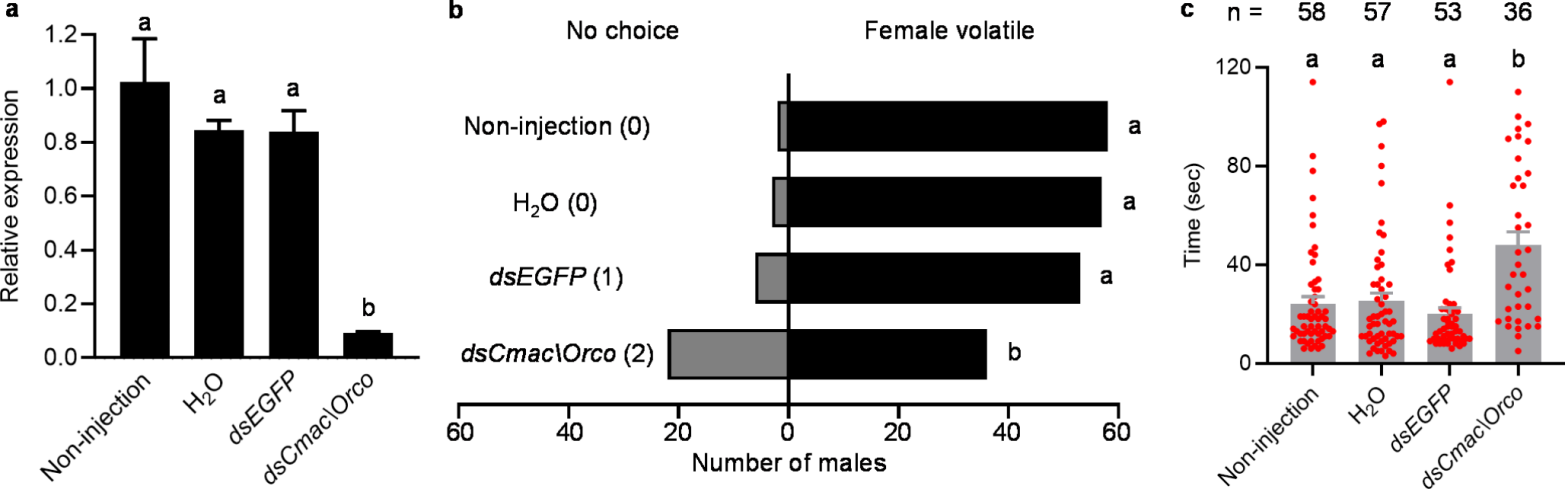
RNAi-mediated knockdown of *Cmac\Orco* in male *C. maculatus.* (**a**) *Cmac\Orco* transcript expression levels of non-injected, H_2_O, *dsEGFP*, and *dsCmac\Orco* microinjected males in the antenna (*n* = 3, one-way ANOVA with Tukey’s test), data are expressed as mean ± standard error of the mean. (**b**) Olfactory responses of the microinjected males to virgin females in Y-tube olfactometer assay (*n* = 60, *χ*^*2*^ test following Fisher’s test for multiple comparison with Bonferroni correction), the numbers in parentheses indicated the number of males attracted to the empty basket. (**c**) Time elapsed until males attracted to the female basket (one-way ANOVA with Tukey-Kramer test), the different letters indicate significant difference at *P* < 0.05

*Statistical analysis.* The relative expression of *Cmac\Orco* transcripts was compared using one-way ANOVA, followed by Tukey’s test (*P* < 0.05). In olfactometer assay, since the males that approached the empty basket were so few (see Fig. 1b), we excluded the numbers from data analysis. The number of male beetles that showed no choice within 2 min and that of approached the female basket were compared using *χ*^2^ test among all treatment groups, followed by Fisher’s test for multiple comparison with Bonferroni correction (*P* < 0.05). The time elapsed until each male made touch the female basket was compared using a one-way ANOVA, followed by the Tukey-Kramer test (*P* < 0.05). All statistical analyses were performed using GraphPad Prism 10 software (GraphPad Software, Boston, MA, USA).

## Results and Discussion

Most seed beetles are the primary pests of stored grains and especially legumes, which are essential protein sources, particularly in developing societies. *C. maculatus* is a common grain pest found globally. Therefore, investigating and elucidating the underlying molecular mechanisms of the odorant-related communication system of *C. maculatus* and disrupting its communication signals would contribute to innovative and sustainable pest management strategies. This strategy proposed the use of a systemic RNAi-based gene knocking down application. However, given that this species completes its life cycle within the seed, pupae was extracted from the seed, injected, and then maintained in a plastic cell culture dish until they matured into adults. Intriguingly, approximately 80% of the pupae extracted from the seeds could eclose, these numbers were comparable to those in the microinjection treatments with and without *dsRNAs* (Table 1), as adults showed normal mating behavior (unpublished observation).

**Table 1:** Numbers of beetles eclosed after microinjection treatment at the pupa stage maintained on 24-well cell culture plate without seeds

		Number of eclosion		Total eclosion rate (%)
Treatments	*N*	Male	Female	
Non-injection	288	110	120	79.9
H_2_O	288	82	104	64.6
*dsEGFP*	288	97	123	76.4
*dsCmac\Orco*	288	90	112	70.1

*Cmac\Orco* knockdown due to *dsCmac\Orco* RNAi silencing was evaluated by qRT-PCR. A ≥ 90% reduction of the *Cmac\Orco* transcript expression was observed in the antenna of injected males compared to that of the non-injected males. Additionally, this was significantly different from the transcripts in the water and *dsEGFP* treatments (F_3,8_ = 20.48, *P* = 0.0004; Fig. 1a).

Non-injected, H_2_O, and *dsEGFP* injected males demonstrated a clear attraction toward the virgin females in the olfactometer assay. However, a significant reduction in the attraction of *dsCmac\Orco* injected males was observed (*χ*^2^ = 38.2, df = 3, P < 0.0001; Fig. 1b). The number of *dsCmac\Orco* injected males with ‘no choice’ was increased, this observation is similar to that of the *dsOrco*-mediated knock down of the red palm weevil, *R. ferrugineus*, as they presented significantly impaired responses to the aggregation pheromone (Soffan et al. 2016). To decipher the *dsCmac\Orco* knockdown effect, the individual time elapsed until the male contacted the female-containing basket within 2 min was compared. It was observed that there was a significant time delay for the *dsCmac\Orco* microinjected males (F_3,199_ = 11.40, *P* < 0.0001; Fig. 1c).

The RNAi methodology has the potential for not only gene functional analysis at the laboratory scale but also for pest management programs in the field (Zhu and Palli 2020). Here, the RNAi silencing of *Cmac\Orco* via microinjection was attempted under laboratory conditions. The *dsCmac\Orco* injection to the dorsal abdominal part of *C. maculatus* pupa resulted in a significant reduction in *Cmac\Orco* expression, mediating a decline in the attraction of male *C. maculatus* toward the female. Insect odorant receptors consist of a heterologous complex with Orco (OR-Orco) to bind and sense odorants; thus, functional analysis of the *Orco* gene is important for clarifying pheromonal reception (Stengl and Funk 2013). Taken together, systemic RNAi-mediated gene knockdown in *C. maculatus* was successfully performed.

Additionally, the RNAi-based gene knockdown application of sex pheromone-specific receptors would be useful to clarify the sex pheromone evolutionary mechanism in congeneric beetles. *Callosobruchus* seed beetles use a hybrid sex pheromone system for mating: sex attractant and contact sex pheromones, both of which show saltational structural changes among congeneric species, for example, homologous short-chain fatty acids have been identified from three congeneric species, *C. maculatus*, *C. subinotatus*, and *C. analis*, whereas homosesquiterpene aldehydes have been identified from *C. chinensis* and *C. rhodesianus* as sex attractant pheromones (Shimomura and Ohsawa 2020). The present application is deserving to identify the specific sex pheromone receptor would decipher such the evolutional adaptation on the saltational structural shift. Moreover, the findings of this study present the basis for the development of olfactory system disruption by RNAi-based biopesticides for seed beetle control.

## Data Availability

No datasets were generated or analysed during the current study.
